# Impact of liver tumour burden, alkaline phosphatase elevation, and target lesion size on treatment outcomes with ^177^Lu-Dotatate: an analysis of the NETTER-1 study

**DOI:** 10.1007/s00259-020-04709-x

**Published:** 2020-03-02

**Authors:** Jonathan Strosberg, Pamela L. Kunz, Andrew Hendifar, James Yao, David Bushnell, Matthew H. Kulke, Richard P. Baum, Martyn Caplin, Philippe Ruszniewski, Ebrahim Delpassand, Timothy Hobday, Chris Verslype, Al Benson, Rajaventhan Srirajaskanthan, Marianne Pavel, Jaume Mora, Jordan Berlin, Enrique Grande, Nicholas Reed, Ettore Seregni, Giovanni Paganelli, Stefano Severi, Michael Morse, David C. Metz, Catherine Ansquer, Frédéric Courbon, Adil Al-Nahhas, Eric Baudin, Francesco Giammarile, David Taïeb, Erik Mittra, Edward Wolin, Thomas M. O’Dorisio, Rachida Lebtahi, Christophe M. Deroose, Chiara M. Grana, Lisa Bodei, Kjell Öberg, Berna Degirmenci Polack, Beilei He, Maurizio F. Mariani, Germo Gericke, Paola Santoro, Jack L. Erion, Laura Ravasi, Eric Krenning, on behalf of the NETTER-1 study group

**Affiliations:** 1 Gastrointestinal Department/Neuroendocrine Tumor Division, Moffitt Cancer Center, Tampa, FL USA; 2 Department of Medicine – Med/Oncology, Stanford University Medical Center, Stanford, CA USA; 3 Department of Internal Medicine/Hematology/Oncology, Cedars Sinai Medical Center, Los Angeles, CA USA; 4 Department of Gastrointestinal Medicinal Oncology, University of Texas MD Anderson Cancer Center, Houston, TX USA; 5 Department of Radiology, The University of Iowa, Iowa City, IA USA; 6 Department of Medical Oncology, Dana-Farber Cancer Institute, Boston, MA USA; 7 Department of Nuclear Medicine, Zentralklinik Bad Berka, Bad Berka, Germany; 8 Department of Gastroenterology and Tumour Neuroendocrinology, Royal Free Hospital, London, UK; 9 Division of Gastroenterology and Pancreatology, Hôpital Beaujon, Clichy, France; 10 Department of Clinical Nuclear Medicine, Excel Diagnostics Imaging Clinic, Houston, TX USA; 11 Department of Oncology, Mayo Clinic College of Medicine, Rochester, MN USA; 12 Department of Hepatology, University Hospitals and KU Leuven, Leuven, Belgium; 13 Hematology Oncology Division, Robert H. Lurie Comprehensive Cancer Center, Chicago, IL USA; 14 Department of Gastroenterology and General Internal Medicine, King’s College Hospital – NHS Foundation Trust, London, UK; 15 Division of Hepatology and Gastroenterology, Charite-Universitätsmedizin Berlin, Berlin, Germany; 16 Department of Nuclear Medicine, Hospital Universitari de Bellvitge, Barcelona, Spain; 17 Department of Medicine, Vanderbilt University Medical Center, Nashville, TN USA; 18 Department of Medical Oncology, MD Anderson Cancer Center, Madrid, Spain; 19 Department of Medical Oncology, Beatson Oncology Centre, Glasgow, UK; 20 Department of Nuclear Medicine Therapy and Endocrinology, Fondazione Istituto di Ricovero e Cura a Carattere Scientifico Istituto Nazionale dei Tumori, Milan, Italy; 21 Department of Medical Oncology, Istituto Scientifico Romagnolo per lo Studio e la Cura dei Tumori (IRST) IRCCS, Meldola, Italy; 22 Department of Surgery, Duke University Medical Center, Durham, NC USA; 23 GI Division, Hospital of the University of Pennsylvania, Philadelphia, PA USA; 24 Nuclear Medicine Department, Hôtel Dieu, University Hospital, Nantes, France; 25 Medical Imaging, Oncology University Institut Claudius Regaud, Toulouse, France; 26 Division of Imaging and Interventional Radiology, Imperial College London, London, UK; 27 Department of Endocrine Oncology and Nuclear Medicine, Institut Gustave Roussy, Villejuif, France; 28 Department of Nuclear Sciences and Applications, International Atomic Energy Agency, Vienna, Austria; 29 Department of Nuclear Medicine, Hôpital de la Timone, Marseille, France; 30 Department of Nuclear Medicine, Oregon Health & Science University, Portland, OR USA; 31 Department of Medicine, Hematology and Medical Oncology, Icahn School of Medicine at Mount Sinai, New York, NY USA; 32 Department of Internal Medicine, The University of Iowa, Iowa City, IA USA; 33 Department of Nuclear Medicine, Royal Free Hospital, London, UK; 34 Nuclear Medicine Department, University Hospitals and KU Leuven, Leuven, Belgium; 35 Division of Nuclear Medicine, Istituto Europeo di Oncologia, Milan, Italy; 36 Department of Nuclear Medicine, Memorial Sloan Kettering Cancer Center, New York, NY USA; 37 Department of Endocrine Oncology, Uppsala University Hospital, Uppsala, Sweden; 38 Department of Medical Information, Advanced Accelerator Applications, a Novartis Company, Geneva, Switzerland; 39 Advanced Accelerator Applications, a Novartis Company, Geneva, Switzerland; 40 Research and Development, Advanced Accelerator Applications, a Novartis Company, Geneva, Switzerland; 41 Department of Clinical Development, Advanced Accelerator Applications, a Novartis Company, Geneva, Switzerland; 42 Department of Nuclear Medicine, Cyclotron Rotterdam BV, Erasmus University Medical Center, Rotterdam, Netherlands

**Keywords:** ^177^Lu-Dotatate, Liver tumour burden, NETTER-1, Neuroendocrine tumour, Octreotide

## Abstract

**Purpose:**

To assess the impact of baseline liver tumour burden, alkaline phosphatase (ALP) elevation, and target lesion size on treatment outcomes with ^177^Lu-Dotatate.

**Methods:**

In the phase 3 NETTER-1 trial, patients with advanced, progressive midgut neuroendocrine tumours (NET) were randomised to 177Lu-Dotatate (every 8 weeks, four cycles) plus octreotide long-acting release (LAR) or to octreotide LAR 60 mg. Primary endpoint was progression-free survival (PFS). Analyses of PFS by baseline factors, including liver tumour burden, ALP elevation, and target lesion size, were performed using Kaplan-Meier estimates; hazard ratios (HRs) with corresponding 95% CIs were estimated using Cox regression.

**Results:**

Significantly prolonged median PFS occurred with ^177^Lu-Dotatate versus octreotide LAR 60 mg in patients with low (< 25%), moderate (25–50%), and high (> 50%) liver tumour burden (HR 0.187, 0.216, 0.145), and normal or elevated ALP (HR 0.153, 0.177), and in the presence or absence of a large target lesion (diameter > 30 mm; HR, 0.213, 0.063). Within the ^177^Lu-Dotatate arm, no significant difference in PFS was observed amongst patients with low/moderate/high liver tumour burden (*P* = 0.7225) or with normal/elevated baseline ALP (*P* = 0.3532), but absence of a large target lesion was associated with improved PFS (*P* = 0.0222). Grade 3 and 4 liver function abnormalities were rare and did not appear to be associated with high baseline liver tumour burden.

**Conclusions:**

^177^Lu-Dotatate demonstrated significant prolongation in PFS versus high-dose octreotide LAR in patients with advanced, progressive midgut NET, regardless of baseline liver tumour burden, elevated ALP, or the presence of a large target lesion.

Clinicaltrials.gov: NCT01578239, EudraCT: 2011-005049-11

**Electronic supplementary material:**

The online version of this article (10.1007/s00259-020-04709-x) contains supplementary material, which is available to authorized users.

## Introduction

The liver is the dominant site of metastatic disease amongst patients with stage IV well-differentiated neuroendocrine tumours (NET) [1]. High liver tumour burden has been shown to be a poor prognostic factor in multiple studies [2–8]. In the phase 3 PROMID study (which randomised patients with midgut NET to octreotide long-acting release [LAR] versus placebo), liver tumour burden > 10% was associated with a hazard ratio (HR) for progression of 2.63 on multivariate analysis [2]. Another prognostic factor is serum alkaline phosphatase (ALP) [9–13], which may be elevated with extensive liver involvement and bone metastases [10, 14]. In one series of metastatic gastrointestinal NET, ALP ≥ upper limit of normal (ULN) was associated with a median progression-free survival (PFS) of 10 months versus 33 months with normal ALP (multivariate HR, 2.49, *P =* 0.017) [10].

Tumour size is often considered a prognostic factor for patients treated with radiolabelled somatostatin analogue (SSA) [15]. Lutetium-177 (^177^Lu) is a beta- and gamma-emitting radionuclide [16]. Compared with Yttrium-90 (^90^Y), ^177^Lu has lower maximum and mean beta particle energies and maximum and mean soft-tissue penetration depths of 1.7 and 0.23 mm, respectively [16], considered ideal for treatment of intermediate-sized tumours but hypothesised to be suboptimal for large tumours [15, 17, 18]. However, correlation between tumour size and ^177^Lu effectiveness has not been evaluated in a randomised controlled trial.

To assess the impact of these potential prognostic and predictive factors on ^177^Lu-Dotatate efficacy and toxicity, we conducted a post hoc analysis of the NETTER-1 trial, the only prospective phase 3 study of a radiolabelled SSA [19]. In NETTER-1, 231 patients with progressive midgut NET were randomised to ^177^Lu-Dotatate every 8 weeks for four cycles, or high-dose octreotide LAR 60 mg every 4 weeks. At the time of primary endpoint data analysis (24 July 2015), median PFS was not reached (NR) in the ^177^Lu-Dotatate arm and was 8.4 months in the control arm (HR 0.21; 95% CI 0.13–0.33) [19]. Health-related QOL analysis (30 June 2016) demonstrated significant improvement in time to decline (TTD) with ^177^Lu-Dotatate in the clinically relevant domains of global health status, physical functioning, role functioning, diarrhoea, pain, and fatigue [20].

We assessed the impact of baseline liver tumour burden on ^177^Lu-Dotatate treatment efficacy outcomes (PFS), TTD in QOL, and hepatic toxicity rates. We evaluated the predictive and prognostic power of elevated ALP, whether presence of ≥ 1 target lesion >3 cm in diameter impacted PFS benefit with ^177^Lu-Dotatate, and whether baseline tumour size correlated inversely with tumour shrinkage rates.

## Methods

### NETTER-1 key eligibility criteria and study design

Eligible patients were aged ≥ 18 years with locally advanced or metastatic, low-, or intermediate-grade (Ki-67 ≤ 20%) NET originating in the midgut with radiologic disease progression (according to Response Evaluation Criteria in Solid Tumours version 1.1 over ≤3 years) while receiving a standard dose of octreotide. All target lesions were required to be somatostatin-receptor-positive. Hepatic exclusion criteria were total bilirubin > 3× ULN and serum albumin ≤ 3.0 g/dL, unless prothrombin time was within normal range.

Patients were randomised to four cycles of ^177^Lu-Dotatate (administered every 8 weeks) along with intramuscular (IM) octreotide LAR 30 mg every 8 weeks (followed by maintenance octreotide LAR 30 mg every 4 weeks) or to high-dose octreotide LAR 60 mg every 4 weeks. Patients were stratified by highest tumour uptake on somatostatin receptor scintigraphy and by duration of prior treatment with constant-dose octreotide LAR (≤ 6 or > 6 months).

The trial protocol was approved by the institutional review board or independent ethics committee at each institution. The trial was performed in accordance with the principles of the Declaration of Helsinki, International Conference on Harmonisation Good Clinical Practice guidelines, and all applicable regulations. All patients provided written informed consent.

### PFS by extent of liver tumour burden

Baseline liver tumour burden was estimated by blinded central radiology review (Keosys, Saint Herblain, France) and categorised into subgroups of low (< 25%), moderate (25–50%), or high (> 50%) tumour burden according to liver tumour volume divided by total liver volume by computed tomography (CT) or magnetic resonance imaging (MRI). The thresholds chosen were similar to those described in prior phase 3 studies evaluating SSAs in NETs [2, 21].

PFS curves for each treatment arm and median PFS with corresponding 95% CIs were generated using Kaplan-Meier estimates, stratified by liver tumour burden, and the log-rank test was used for within–treatment arm comparisons of PFS. HRs with corresponding 95% CIs and *P-*values were estimated using a Cox regression model with randomised treatment, liver tumour burden at baseline and liver tumour burden × randomised treatment interaction term as covariates. The primary data analysis cutoff was 24 July 2015.

### PFS by baseline ALP

PFS curves were generated for each treatment arm, stratified by baseline ALP (normal, or > ULN, based on institutional ULN), and the log-rank test was used for within–treatment arm PFS comparisons. HRs with corresponding 95% CIs and *P-*values were generated using the methodology described above.

### PFS by presence or absence of a large lesion

Patients were stratified into two subgroups based on the presence or absence of at least one target lesion >30 mm in diameter at any body site on CT or MRI at baseline. This approximate size threshold has been described in previous literature as distinguishing ‘large’ tumours from smaller ones in animal studies of peptide receptor radionuclide therapy (PRRT) [18, 22]. PFS curves were generated for each treatment arm, stratified by the presence or absence of large target tumour, and the log-rank test was used for within–treatment arm comparisons of PFS. HRs with corresponding 95% CIs and *P-*values were generated using the methodology described above.

### Liver lesion shrinkage by baseline liver lesion size

A mixed model repeated measures (MMRM) analysis included study visit, baseline tumour size (≤ 30 mm and > 30 mm), and baseline tumour size × study visit interaction as fixed effects, and was used to evaluate the effect of baseline tumour size on least squares mean percentage change in tumour size from baseline to week 72 (data cutoff, 30 June 2016).

### Hepatic toxicity by extent of liver tumour burden

Assessment of grade 3 or 4 liver function test (LFT) abnormalities (aspartate aminotransferase [AST], alanine aminotransferase [ALT], ALP, albumin, and bilirubin) was stratified by tumour burden categories described above. The analysis comprised all patients who underwent randomisation and received at least one dose of trial treatment (data cutoff, 30 June 2016). Adverse events in NETTER-1 were graded according to the National Cancer Institute Common Terminology Criteria for Adverse Events, version 4.02.

### QOL by extent of liver tumour burden

TTD of QOL (data cutoff, 30 June 2016) was defined as the time from randomisation to first deterioration ≥ 10 points (100-point scale) compared with baseline on EORTC QLQ-C30 and GI-NET21. TTD was estimated using Kaplan-Meier methodology and stratified by liver tumour burden subgroup: low (< 25%) or moderate to high (≥ 25%).

## Results

In total, 231 patients (117 ^177^Lu-Dotatate patients, 114 high-dose octreotide patients) were enrolled in NETTER-1; 223 received at least one dose of study drug and were eligible for safety analysis (see Fig. **S1** in the Supplementary material). At the time of the primary PFS analysis, 229 patients were enrolled. Most had liver metastases at baseline (98/116 [84.5%] and 94/113 [83.2%] in the ^177^Lu-Dotatate and octreotide arms, respectively). **Supplementary Table****S1** summarizes the distribution of patients stratified by liver tumour burden, ALP elevation, and presence of a large target lesion at baseline.

### PFS by extent of liver tumour burden

**Fig. 1:**
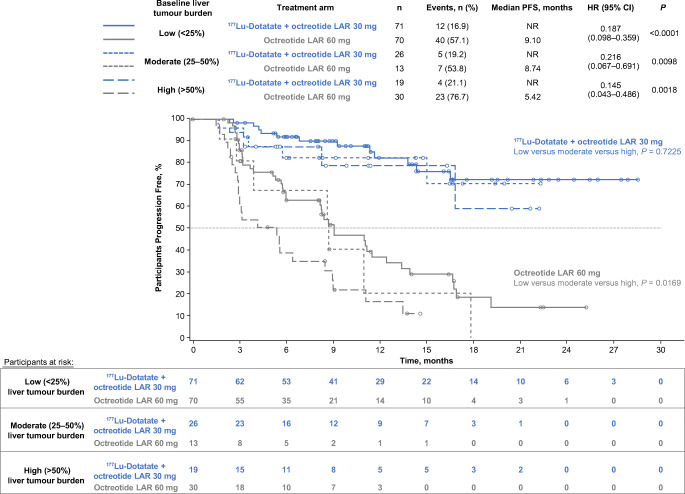
Kaplan-Meier analysis of progression-free survival by treatment arm (patients randomised to four cycles of peptide receptor radionuclide therapy with ^177^Lu-Dotatate + octreotide LAR 30 mg or octreotide LAR 60 mg) and baseline extent of liver tumour burden (low [< 25%], moderate [25–50%], or high [> 50%]). Liver tumour burden is calculated according to liver tumour volume divided by total liver volume by computed tomography or magnetic resonance imaging. Data cutoff: 24 July 2015. HRs with corresponding 95% CIs and *P-*values were estimated using a Cox regression model with randomised treatment, liver tumour burden at baseline, and liver tumour burden × randomised treatment interaction term as covariates. Log-rank test used for within-treatment arm comparisons of PFS. CI: confidence interval, HR: hazard ratio, LAR: long-acting release, NR: not reached, PFS: progression-free survival

Statistically and clinically significant prolongation of PFS with ^177^Lu-Dotatate was observed in patients with low, moderate, and high liver tumour burden, with nearly identical HRs for progression or death across all prognostic groups (Fig. 1). Median PFS was NR in the ^177^Lu-Dotatate arm versus 9.1 months in the high-dose octreotide arm (HR 0.19; *P* < 0.0001) in those with low burden; NR versus 8.7 months in those with moderate burden (HR 0.22; *P =* 0.0098); and NR versus 5.4 months in those with high burden (HR 0.15; *P =* 0.0018).

Within the ^177^Lu-Dotatate arm, no significant difference in PFS was observed with low, moderate, or high baseline tumour burden (log-rank *P =* 0.7225). However, within the high-dose octreotide arm, there was a significant correlation between liver tumour burden and PFS, with median PFS of 9.1, 8.7, and 5.4 months for low, moderate, and high burdens, respectively (log-rank *P =* 0.0169).

### PFS by normal or elevated ALP

**Fig. 2:**
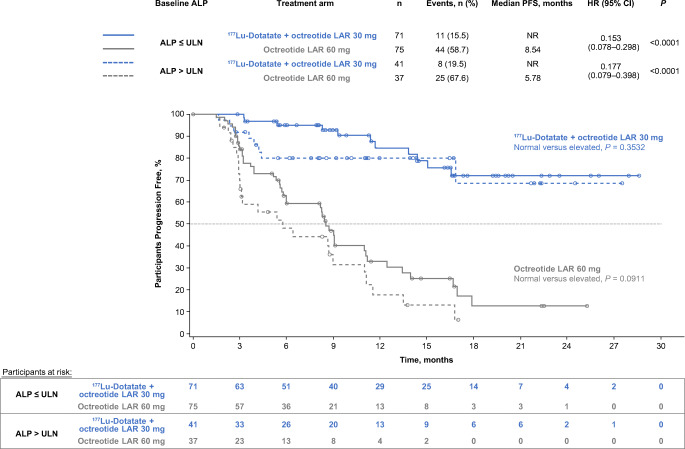
Kaplan-Meier analysis of progression-free survival by treatment arm (patients randomised to four cycles of peptide receptor radionuclide therapy with ^177^Lu-Dotatate + octreotide LAR 30 mg or octreotide LAR 60 mg) and baseline normal (≤ ULN) or elevated (> ULN) alkaline phosphatase levels (based on institutional ULN). Data cutoff: 24 July 2015. One-hundred twelve patients in either treatment arm had evaluable baseline ALP levels and were included in this analysis. HRs with corresponding 95% CIs and *P-*values were estimated using a Cox regression model with randomised treatment, alkaline phosphatase level, and alkaline phosphatase level × randomised treatment interaction term as covariates. Log-rank test was used for within-treatment arm comparisons of PFS. ALP: alkaline phosphatase, CI: confidence interval, HR: hazard ratio, LAR: long-acting release, NR: not reached, PFS: progression-free survival, ULN: upper limit of normal

In each treatment arm, 112 patients had evaluable baseline ALP. Statistically and clinically significant prolongation of PFS with ^177^Lu-Dotatate was observed amongst patients with normal and elevated baseline ALP, with nearly identical HRs for progression or death in both prognostic groups (Fig. 2), as reported in the original subgroup analysis of the NETTER-1 study [19]. Median PFS was NR in the ^177^Lu-Dotatate arm versus 8.5 months in the high-dose octreotide arm (HR 0.15; *P* < 0.0001) in the normal ALP group and NR versus 5.8 months (HR 0.18; *P* < 0.0001) in the elevated baseline ALP group**.**

No significant difference in PFS was observed amongst patients with normal versus elevated ALP in the ^177^Lu-Dotatate (log-rank *P =* 0.3532) or high-dose octreotide arm (log-rank *P =* 0.0911).

### PFS by presence of a large target lesion

**Fig. 3:**
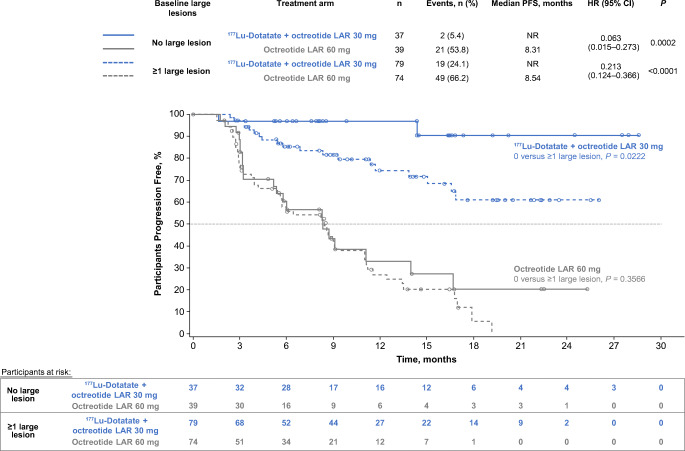
Kaplan-Meier analysis of progression-free survival by treatment arm (patients randomised to four cycles of peptide receptor radionuclide therapy with ^177^Lu-Dotatate + octreotide LAR 30 mg or octreotide LAR 60 mg) and presence or absence of at least one large (> 30 mm diameter) target lesion at any site of the body at baseline imaging with computed tomography or magnetic resonance imaging. Data cutoff: 24 July 2015. HRs with corresponding 95% CIs and *P-*values were estimated using a Cox regression model with randomised treatment, presence/absence of large target lesion, and presence/absence of large target lesion × randomised treatment interaction term as covariates. Log-rank test was used for within–treatment arm comparisons of PFS. CI: confidence interval, HR: hazard ratio, LAR: long-acting release, NR: not reached, PFS: progression-free survival

Amongst target lesions in patients within the ^177^Lu-Dotatate arm, 128 large tumours (>30 mm diameter) were identified, of which 89 (70%) were liver tumours; in the high-dose octreotide arm, 134 large tumours were identified; 93 (69%) were liver tumours. Regardless of presence or absence of a large baseline lesion, median PFS was significantly prolonged amongst patients treated with ^177^Lu-Dotatate versus high-dose octreotide (Fig. 3). The benefit was particularly pronounced amongst patients with no large target baseline lesion: median PFS was NR in the ^177^Lu-Dotatate arm versus 8.3 months in the high-dose octreotide arm (HR 0.063; *P =* 0.0002). However, there was also clinically and statistically significant benefit of ^177^Lu-Dotatate amongst patients with ≥ 1 large target tumour; median PFS was NR in the ^177^Lu-Dotatate arm versus 8.5 months in the high-dose octreotide arm (HR 0.21; *P* < 0.0001).

The presence or absence of a large baseline lesion did not impact the PFS of patients receiving high-dose octreotide (median PFS, 8.5 versus 8.3 months; log-rank *P =* 0.3566). However, absence of a large target lesion was associated with improved PFS in the ^177^Lu-Dotatate arm (log-rank *P =* 0.0222), although median PFS was NR in both groups.

### Decrease in target liver tumour diameter stratified by baseline liver tumour size

**Fig. 4:**
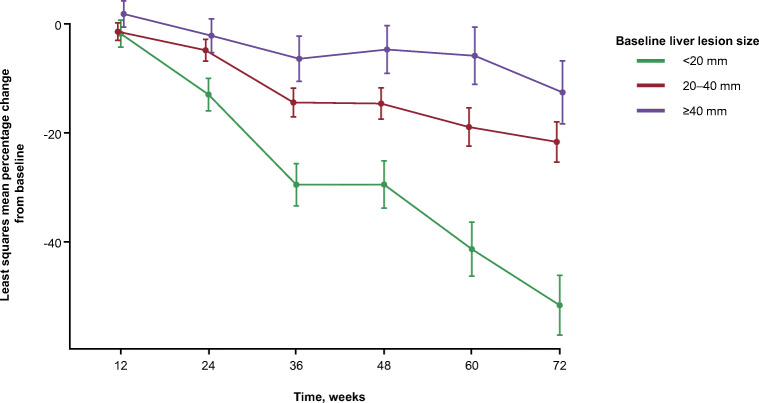
Least squares mean percentage change from baseline in the size of liver lesions at each study visit in the ^177^Lu-Dotatate arm, stratified by baseline liver lesion size. Data cutoff: 30 June 2016. A lesion-based mixed model repeated measures analysis included study visit, baseline target liver lesion size (≤ 30 mm or > 30 mm), and baseline target liver lesion size × study visit interaction as fixed effects

To assess whether baseline liver tumour size correlates with radiographic tumour shrinkage in patients receiving ^177^Lu-Dotatate, we stratified target lesions into two groups based on tumour diameter: ≤ 30 mm and > 30 mm. Changes in measurements at each scanning interval up to 72 weeks were evaluated for each lesion and averaged for each baseline size category (Fig. 4). Tumour size significantly decreased from baseline to week 72 (*P* < 0.0001) regardless of baseline size. At 72 weeks, least squares mean shrinkage was 29% and 14% in the ≤ 30 mm and > 30 mm groups, respectively. There was a significant interaction of baseline tumour size by time of visit (*P =* 0.0085) within the ^177^Lu-Dotatate-treated group, indicating that liver tumour size shrinkage over time differs by baseline size.

### TTD in QOL stratified by baseline liver tumour burden

In patients with low tumour burden (< 25%), median TTD of global health status was 28.8 months in the ^177^Lu-Dotatate arm versus 6.1 months in the high-dose octreotide arm (HR 0.376; *P =* 0.0022). In patients with moderate/high tumour burden (≥ 25%), the median TTD of global health status was NR in the ^177^Lu-Dotatate versus 6.0 months in the high-dose octreotide arm (HR 0.45; *P =* 0.0868). The median TTD of other clinically relevant QOL domains stratified by tumour burden are shown in **Supplementary Table****S2**.

### Analysis of hepatic toxicity by extent of baseline liver tumour burden

Grade 3 and 4 LFT abnormalities were rare and did not appear to be associated with high baseline liver tumour burden in either arm (Table 1). Because of the very low frequency of clinically significant toxicity in both arms, a comparative statistical test was not performed.

**Table. 1:** Frequency of grade 3 or 4 liver function test abnormalities in the safety population by treatment arm (patients randomised to four cycles of peptide receptor radionuclide therapy with ^177^Lu-Dotatate + octreotide LAR 30 mg or octreotide LAR 60 mg) and baseline liver tumour burden (low [< 25%], moderate [25–50%], or high [> 50%]). Liver tumour burden is calculated according to liver tumour volume divided by total liver volume by computed tomography or magnetic resonance imaging

Baseline liver tumour burden	Treatment	No. of Patients	Grade 3 or 4 Liver function test abnormalities, no. of patients				
			↑ AST	↑ ALT	↑ ALP	↓ Albumin	↑ Bilirubin
<25%	^177^Lu-Dotatate + octreotide LAR 30 mg	68	2	3	4	0	1
	Octreotide LAR 60 mg	70	0	0	3	0	0
25–50%	^177^Lu-Dotatate + octreotide LAR 30 mg	25	0	0	0	0	1
	Octreotide LAR 60 mg	12	0	0	0	0	0
>50%	^177^Lu-Dotatate + octreotide LAR 30 mg	18	3	1	2	0	0
	Octreotide LAR 60 mg	30	0	0	7	0	0

Data cutoff: 30 June 2016

ALP: alkaline phosphatase, ALT: alanine aminotransferase, AST: aspartate aminotransferase, LAR: long-acting release

## Discussion

The impact of liver tumour burden and largest tumour size on outcomes with ^177^Lu-Dotatate has not been well established, partly owing to lack of randomised studies, which are often necessary to identify predictive factors. Two retrospective studies of ^177^Lu-Dotatate have demonstrated that tumour burden ≥ 25% is associated with a shorter median OS in multivariate analyses (HR 2.9 and 2.1, respectively); however, the relationship with PFS was not investigated [5, 6]. Our analysis demonstrates that high tumour burden does not predict diminished PFS benefit from ^177^Lu-Dotatate versus high-dose octreotide. Indeed, the HR for PFS benefit in the high tumour burden group was nearly identical to the benefit in the low burden cohort. When evaluating each treatment arm separately, high tumour burden was a negative prognostic factor for PFS in the high-dose octreotide arm but did not correlate with negative outcomes in the ^177^Lu-Dotatate arm, suggesting that ^177^Lu-Dotatate may mitigate the negative impact of tumour burden.

Similar findings were observed with ALP elevation as with tumour burden, which is consistent with the association of ALP with tumour burden [10]. The HR for PFS benefit with ^177^Lu-Dotatate versus high-dose octreotide in the high ALP group was nearly identical to the benefit in the normal ALP group. A study of patients treated with ^177^Lu-Dotatate has demonstrated ALP elevation (> 120 IU/L) to be a negative prognostic factor in terms of OS, but did not assess PFS [9].

In this study, presence or absence of a large (> 30 mm) target lesion did not impact the PFS of patients receiving high-dose octreotide (median PFS 8.3 versus 8.5 months, respectively). This suggests that the effect of octreotide is independent of tumour size. Patients lacking a large target lesion had a particularly pronounced PFS benefit with ^177^Lu-Dotatate versus high-dose octreotide, with a 94% improvement in risk of progression or death (HR 0.06). PFS benefit with ^177^Lu-Dotatate versus high-dose octreotide was also seen with at least one large target lesion (HR 0.21). However, in those receiving ^177^Lu-Dotatate, absence of a large target lesion was associated with improved PFS. Mean tumour shrinkage with ^177^Lu-Dotatate correlated with baseline tumour size, being highest in target lesions ≤ 30 mm. These outcomes indicate the effectiveness of ^177^Lu-Dotatate across a spectrum of tumour sizes but also suggest that its effectiveness is particularly high in smaller tumours. Randomized trials are necessary to prove or disprove the hypothesis that longer-range radionuclides (e.g, ^90^Y) should be used in combination or as an alternative to ^177^Lu-based PRRT in patients with large tumours.

The QOL findings suggest that ^177^Lu-Dotatate has a clinically relevant beneficial impact on overall QOL as well as on specific NET-related symptoms regardless of tumour burden. However, when stratified by tumour burden, most QOL results were not significant owing to the small number of patients in each cohort (data not shown).

Concerns exist regarding the safety of ^177^Lu-Dotatate in patients with high tumour burden owing to the potential for radiation hepatitis. Data from NETTER-1 did not validate this hypothesis. LFT elevations were rare and did not appear to correlate with baseline tumour burden. It is important to note, however, that safety findings in patients with tumour burden > 50% do not necessarily imply that treatment is equally safe in patients with extreme tumour burden (e.g., > 90%). A limitation of this study is that central readers did not specify the patients with extreme tumour burden (> 90%), and therefore no specific safety analysis in that subgroup was possible.

In summary, ^177^Lu-Dotatate demonstrated significant prolongation in PFS versus high-dose octreotide in patients with advanced, progressive midgut NET, regardless of baseline liver tumour burden, elevated ALP, or presence of a large target lesion. ^177^Lu-Dotatate is effective across a spectrum of tumour sizes, but its effectiveness is particularly high in smaller tumours, potentially supporting early treatment in patients with progressive disease. Clinically relevant LFT abnormalities were rare and were not associated with high baseline liver tumour burden.

## Electronic supplementary material

ESM 1 (DOCX 15 kb)

ESM 2 (DOCX 15 kb)

ESM 3 (DOCX 16 kb)

ESM 4 (DOCX 282 kb)

ESM 5 (DOCX 16 kb)

## References

[1] Riihimaki M, Hemminki A, Sundquist K (2016). The epidemiology of metastases in neuroendocrine tumors. Int J Cancer.

[2] Rinke A, Müller HH, Schade-Brittinger C (2009). Placebo-controlled, double-blind, prospective, randomized study on the effect of octreotide LAR in the control of tumor growth in patients with metastatic neuroendocrine midgut tumors: a report from the PROMID study group. J Clin Oncol.

[3] Rinke A, Wittenberg M, Schade-Brittinger C (2017). Placebo controlled, double blind, prospective, randomized study on the effect of octreotide LAR in the control of tumor growth in patients with metastatic neuroendocrine midgut tumors (PROMID): results on long term survival. Neuroendocrinology.

[4] Yalchin M, Oliveira A, Theocharidou E (2017). The impact of radiological response to peptide receptor radionuclide therapy on overall survival in patients with metastatic midgut neuroendocrine tumors. Clin Nucl Med.

[5] Ezziddin S, Khalaf F, Vanezi M (2014). Outcome of peptide receptor radionuclide therapy with 177Lu-octreotate in advanced grade 1/2 pancreatic neuroendocrine tumours. Eur J Nucl Med Mol Imaging.

[6] Ezziddin S, Attassi M, Yong-Hing CJ (2014). Predictors of long-term outcome in patients with well-differentiated gastroenteropancreatic neuroendocrine tumors after peptide receptor radionuclide therapy with 177Lu-octreotate. J Nucl Med.

[7] Panzuto F, Merola E, Pavel ME (2017). Stage IV gastro-entero-pancreatic neuroendocrine neoplasms: a risk score to predict clinical outcome. Oncologist.

[8] Foulfoin M, Graillot E, Adham M (2017). Treatment of metastatic pancreatic neuroendocrine tumors: relevance of ENETS 2016 guidelines. Endocr Relat Cancer.

[9] Brabander T, van der Zwan WA, Teunissen JJM et al. Long-Term Efficacy, Survival, and Safety of [(177)Lu-DOTA(0),Tyr(3)]octreotate in Patients with Gastroenteropancreatic and Bronchial Neuroendocrine Tumors. Clin Cancer Res 2017; 23: 4617–4624. 10.1158/1078-0432.CCR-16-2743 28428192

[10] Andriantsoa M, Hoibian S, Autret A (2017). An elevated serum alkaline phosphatase level in hepatic metastases of grade 1 and 2 gastrointestinal neuroendocrine tumors is unusual and of prognostic value. PLoS One.

[11] Fairweather M, Swanson R, Wang J (2017). Management of neuroendocrine tumor liver metastases: long-term outcomes and prognostic factors from a large prospective database. Ann Surg Oncol.

[12] Jimenez-Fonseca P, Krug S, Tamagno G, et al. Identifying prognostic factors for well-differentiated metastatic pancreatic neuroendocrine tumours (pNETs): a retrospective international multicenter cohort study. Neuroendocrinology. 2018. 10.1159/000492223**[Epub ahead of print]**. 10.1159/000492223 30025389

[13] Clancy TE, Sengupta TP, Paulus J (2006). Alkaline phosphatase predicts survival in patients with metastatic neuroendocrine tumors. Dig Dis Sci.

[14] Putzer D, Gabriel M, Henninger B (2009). Bone metastases in patients with neuroendocrine tumor: 68Ga-DOTA-Tyr3-octreotide PET in comparison to CT and bone scintigraphy. J Nucl Med.

[15] Bodei L, Cremonesi M, Grana CM (2012). Yttrium-labelled peptides for therapy of NET. Eur J Nucl Med Mol Imaging.

[16] Bodei L, Mueller-Brand J, Baum RP (2013). The joint IAEA, EANM, and SNMMI practical guidance on peptide receptor radionuclide therapy (PRRNT) in neuroendocrine tumours. Eur J Nucl Med Mol Imaging.

[17] Hicks RJ, Kwekkeboom DJ, Krenning E (2017). ENETS consensus guidelines for the standards of care in neuroendocrine neoplasia: peptide receptor radionuclide therapy with radiolabeled somatostatin analogues. Neuroendocrinology.

[18] de Jong M, Breeman WA, Valkema R (2005). Combination radionuclide therapy using 177Lu- and 90Y-labeled somatostatin analogs. J Nucl Med.

[19] Strosberg J, El-Haddad G, Wolin E (2017). Phase 3 trial of 177Lu-Ddotatate for midgut neuroendocrine tumors. N Engl J Med.

[20] Strosberg J, Wolin E, Chasen B (2018). Health-related quality of life in patients with progressive midgut neuroendocrine tumors treated with 177Lu-dotatate in the phase III NETTER-1 trial. J Clin Oncol.

[21] Caplin ME, Pavel M, Cwikla JB (2014). Lanreotide in metastatic enteropancreatic neuroendocrine tumors. N Engl J Med.

[22] de Jong M, Breeman WA, Bernard BF (2001). Tumor response after [(90)Y-DOTA(0),Tyr(3)]octreotide radionuclide therapy in a transplantable rat tumor model is dependent on tumor size. J Nucl Med.

