# Supplementary material for: Impact of liver tumour burden, alkaline phosphatase elevation, and target lesion size on treatment outcomes with 177Lu-Dotatate: an analysis of the NETTER-1 study
Source: Eur J Nucl Med Mol Imaging. 2020 Mar 2;47(10):2372–82. doi: 10.1007/s00259-020-04709-x (PMC7396396; doi:10.1007/s00259-020-04709-x)
Supplement: Supplementary file 3 — (DOCX 16 kb) [file 259_2020_4709_MOESM3_ESM.docx]

**Impact of Liver Tumour Burden, Alkaline Phosphatase Elevation, and Target Lesion Size on Treatment Outcomes With ^177^Lu-Dotatate: An Analysis of the NETTER-1 Study**

Jonathan Strosberg, Pamela L. Kunz, Andrew Hendifar, James Yao, David Bushnell, Matthew H. Kulke, Richard P. Baum, Martyn Caplin, Philippe Ruszniewski, Ebrahim Delpassand, Timothy Hobday, Chris Verslype, Al Benson, Rajaventhan Srirajaskanthan, Marianne Pavel, Jaume Mora, Jordan Berlin, Enrique Grande, Nicholas Reed, Ettore Seregni, Giovanni Paganelli, Stefano Severi, Michael Morse, David C. Metz, Catherine Ansquer, Frédéric Courbon, Adil Al-Nahhas, Eric Baudin, Francesco Giammarile, David Taïeb, Erik Mittra, Edward Wolin, Thomas M. O’Dorisio, Rachida Lebtahi, Christophe M. Deroose, Chiara M. Grana, Lisa Bodei, Kjell Öberg, Berna Degirmenci Polack, Beilei He, Maurizio F. Mariani, Germo Gericke, Paola Santoro, Jack L. Erion, Laura Ravasi, Eric Krenning; on behalf of the NETTER-1 study group.

**Correspondence:**

Dr Jonathan Strosberg

H Lee Moffitt Cancer Center and Research Institute

12902 Magnolia Dr

Tampa, FL 33612

Phone: 813-745-6650

E-mail: [jonathan.strosberg@moffitt.org](mailto:jonathan.strosberg@moffitt.org)

**Supplementary Table S3.** Median time to decline in QOL domains in patients with low (<25%) or moderate to high (≥25%) baseline liver tumour burden

| **Baseline Liver Tumour Burden** | **Treatment Arm** | **No. of Patients** | **Median TTD, months** | | | | | | |
| --- | --- | --- | --- | --- | --- | --- | --- | --- | --- |
|  |  |  | **EORTC QLQ-C30 Domains** | | | | | | **GI.NET21 Domain** |
|  |  |  | **Global Health Status** | **Physical Functioning** | **Role Functioning** | **Diarrhea** | **Fatigue** | **Pain** | **Endocrine** |
| **<25%** | ^177^Lu-Dotatate + octreotide LAR 30 mg | 71 | 28.81 | 25.20 | 14.72 | NR | 8.97 | 13.01 | 11.76 |
|  | Octreotide LAR 60 mg | 70 | 6.11 | 11.47 | 11.30 | NR | 5.98 | 11.20 | 14.23 |
|  | Hazard ratio  (95% CI) | | 0.376  (0.196–0.720) | 0.512  (0.264–0.994) | 0.651  (0.362–1.170) | 0.438  (0.206–0.930) | 0.550  (0.318–0.951) | 0.663  (0.367-1.199) | 0.967  (0.512-1.827) |
| **≥25%** | ^177^Lu-Dotatate + octreotide LAR 30 mg | 46 | NR | NR | NR | NR | 5.88 | NR | NR |
|  | Octreotide LAR 60 mg | 44 | 5.98 | 11.56 | 11.56 | 11.89 | 5.78 | 11.10 | 16.72 |
|  | Hazard ratio  (95% CI) | | 0.453  (0.178–1.152) | 0.526  (0.207– 1.335) | 0.425  (0.161–1.120) | 0.500  (0.192–1.301) | 0.764  (0.366–1.596) | 0.411  (0.156–1.080) | 0.751  (0.255–2.210) |

CI: confidence interval, EORTC QLQ-C30: European Organisation for Research and Treatment of Cancer Core Quality of Life Questionnaire, NR: not reached, TTD, time to decline.

Data cutoff date: 30 June 2016.
