# Supplementary material for: Impact of liver tumour burden, alkaline phosphatase elevation, and target lesion size on treatment outcomes with 177Lu-Dotatate: an analysis of the NETTER-1 study
Source: Eur J Nucl Med Mol Imaging. 2020 Mar 2;47(10):2372–82. doi: 10.1007/s00259-020-04709-x (PMC7396396; doi:10.1007/s00259-020-04709-x)
Supplement: Supplementary file 4 — (DOCX 282 kb) [file 259_2020_4709_MOESM4_ESM.docx]

**Impact of Liver Tumour Burden, Alkaline Phosphatase Elevation, and Target Lesion Size on Treatment Outcomes With ^177^Lu-Dotatate: An Analysis of the NETTER-1 Study**

Jonathan Strosberg, Pamela L. Kunz, Andrew Hendifar, James Yao, David Bushnell, Matthew H. Kulke, Richard P. Baum, Martyn Caplin, Philippe Ruszniewski, Ebrahim Delpassand, Timothy Hobday, Chris Verslype, Al Benson, Rajaventhan Srirajaskanthan, Marianne Pavel, Jaume Mora, Jordan Berlin, Enrique Grande, Nicholas Reed, Ettore Seregni, Giovanni Paganelli, Stefano Severi, Michael Morse, David C. Metz, Catherine Ansquer, Frédéric Courbon, Adil Al-Nahhas, Eric Baudin, Francesco Giammarile, David Taïeb, Erik Mittra, Edward Wolin, Thomas M. O’Dorisio, Rachida Lebtahi, Christophe M. Deroose, Chiara M. Grana, Lisa Bodei, Kjell Öberg, Berna Degirmenci Polack, Beilei He, Maurizio F. Mariani, Germo Gericke, Paola Santoro, Jack L. Erion, Laura Ravasi, Eric Krenning; on behalf of the NETTER-1 study group.

**Correspondence:**

Dr Jonathan Strosberg

H Lee Moffitt Cancer Center and Research Institute

12902 Magnolia Dr

Tampa, FL 33612

Phone: 813-745-6650

E-mail: [jonathan.strosberg@moffitt.org](mailto:jonathan.strosberg@moffitt.org)

**Supplementary Figure S1.** CONSORT diagram for NETTER-1. LAR: LAR: long-acting release.


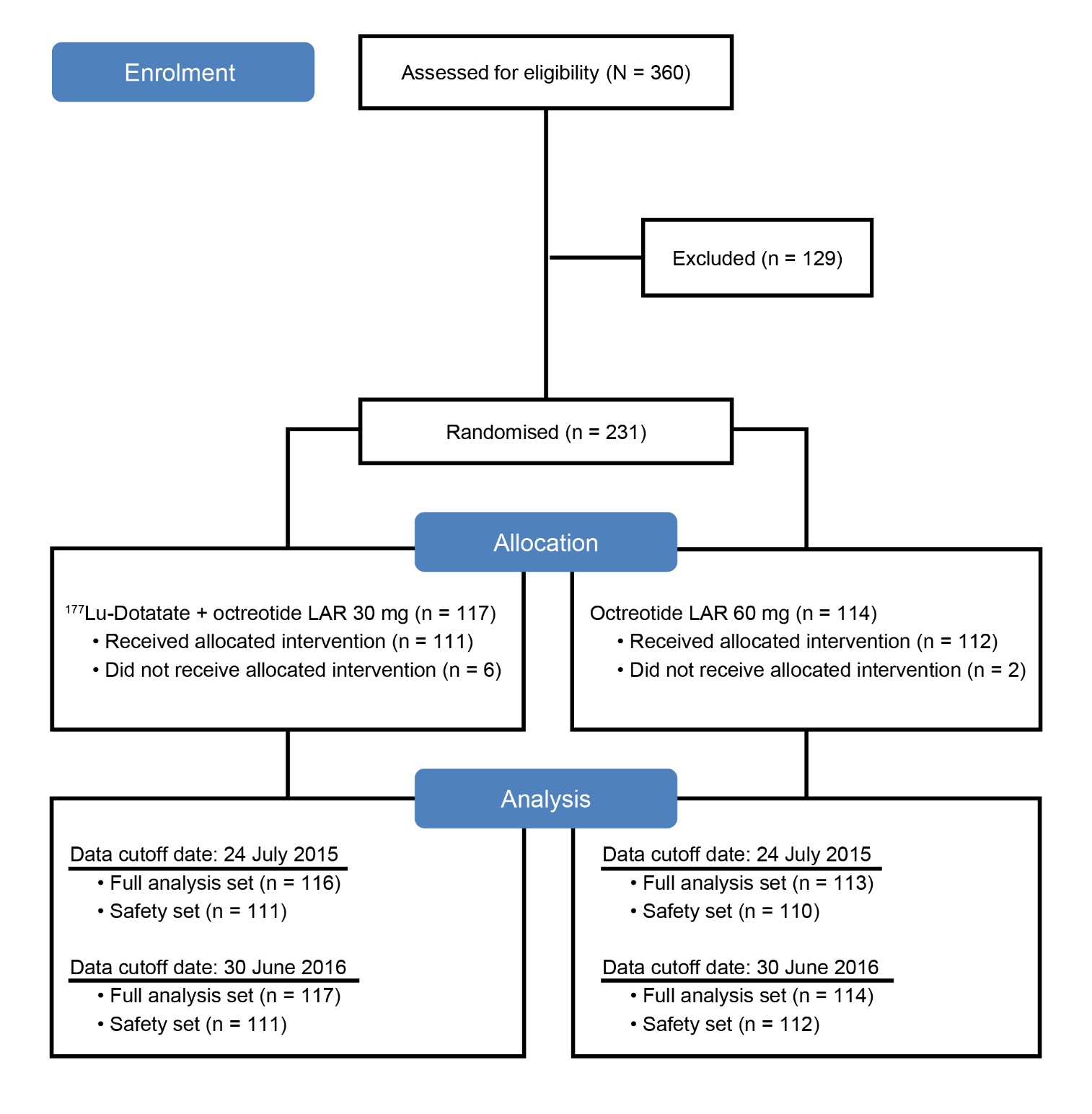


**I**
