# Supplementary material for: Impact of liver tumour burden, alkaline phosphatase elevation, and target lesion size on treatment outcomes with 177Lu-Dotatate: an analysis of the NETTER-1 study
Source: Eur J Nucl Med Mol Imaging. 2020 Mar 2;47(10):2372–82. doi: 10.1007/s00259-020-04709-x (PMC7396396; doi:10.1007/s00259-020-04709-x)
Supplement: Supplementary file 5 — (DOCX 16 kb) [file 259_2020_4709_MOESM5_ESM.docx]

**Impact of Liver Tumour Burden, Alkaline Phosphatase Elevation, and Target Lesion Size on Treatment Outcomes With ^177^Lu-Dotatate: An Analysis of the NETTER-1 Study**

Jonathan Strosberg, Pamela L. Kunz, Andrew Hendifar, James Yao, David Bushnell, Matthew H. Kulke, Richard P. Baum, Martyn Caplin, Philippe Ruszniewski, Ebrahim Delpassand, Timothy Hobday, Chris Verslype, Al Benson, Rajaventhan Srirajaskanthan, Marianne Pavel, Jaume Mora, Jordan Berlin, Enrique Grande, Nicholas Reed, Ettore Seregni, Giovanni Paganelli, Stefano Severi, Michael Morse, David C. Metz, Catherine Ansquer, Frédéric Courbon, Adil Al-Nahhas, Eric Baudin, Francesco Giammarile, David Taïeb, Erik Mittra, Edward Wolin, Thomas M. O’Dorisio, Rachida Lebtahi, Christophe M. Deroose, Chiara M. Grana, Lisa Bodei, Kjell Öberg, Berna Degirmenci Polack, Beilei He, Maurizio F. Mariani, Germo Gericke, Paola Santoro, Jack L. Erion, Laura Ravasi, Eric Krenning; on behalf of the NETTER-1 study group.

**Correspondence:**

Dr Jonathan Strosberg

H Lee Moffitt Cancer Center and Research Institute

12902 Magnolia Dr

Tampa, FL 33612

Phone: 813-745-6650

E-mail: [jonathan.strosberg@moffitt.org](mailto:jonathan.strosberg@moffitt.org)

**NETTER-1 Study Investigators**

**Belgium**: Eric Van Cutsem; **France**: Catherine Ansquer, Eric Baudin, Frederic Courbon, Francesco Giammarile, Philippe Ruszniewski, David Taieb; **Germany**: Richard P. Baum, Marianne Pavel, Klemens Scheidhauer, Matthias Weber; **Italy**: Lisa Bodei, Ernesto Brianzoni, Gianfranco Delle Fave, Maria Chiara Grana, Giuliano Mariani, Guido Rindi, Ettore Seregni, Stefano Severi; **Portugal**: Isabel Azevedo; **Spain**: Enrique Grande, Jaime Mora; **Sweden**: Kjell Öberg, Anders Sundin; **United Kingdom**: Adil Al Nahhas, Martyn Caplin, Nick Freemantle, Ashley Grossman, Prakash Manoharan, Nicholas Reed, Rajaventhan Srirajaskanthan; **USA**: Lowell Anthony, Al B. Benson, Jordan Berlin, David Bushnell, Ebrahim Delpassand, Stanley Garbus, Andrew Hendifar, Timothy Hobday, Matthew Kulke, Pamela Kunz, Larry Kvols, David Metz, Erik Mittra, Michael Morse, Meike Schipper, Jonathan Strosberg, Edward Wolin, James Yao.
